# Focusing X-ray free-electron laser pulses using Kirkpatrick–Baez mirrors at the NCI hutch of the PAL-XFEL

**DOI:** 10.1107/S1600577517016186

**Published:** 2018-01-01

**Authors:** Jangwoo Kim, Hyo-Yun Kim, Jaehyun Park, Sangsoo Kim, Sunam Kim, Seungyu Rah, Jun Lim, Ki Hyun Nam

**Affiliations:** a Pohang Accelerator Laboratory, POSTECH, Pohang, Gyeongbuk 37673, Republic of Korea

**Keywords:** microfocusing, X-ray optics, KB mirror, X-ray free-electron laser, PAL-XFEL, NCI

## Abstract

The Pohang Accelerator Laboratory X-ray Free-Electron Laser (PAL-XFEL) is a recently commissioned X-ray free-electron laser (XFEL) facility that provides intense ultrashort X-ray pulses based on the self-amplified spontaneous emission process. The nano-crystallography and coherent imaging (NCI) hutch with forward-scattering geometry is located at the hard X-ray beamline of the PAL-XFEL and provides opportunities to perform serial femtosecond crystallography and coherent X-ray diffraction imaging. To produce intense high-density XFEL pulses at the interaction positions between the X-rays and various samples, a microfocusing Kirkpatrick–Baez (KB) mirror system that includes an ultra-precision manipulator has been developed. In this paper, the design of a KB mirror system that focuses the hard XFEL beam onto a fixed sample point of the NCI hutch, which is positioned along the hard XFEL beamline, is described. The focusing system produces a two-dimensional focusing beam at approximately 2 µm scale across the 2–11 keV photon energy range. XFEL pulses of 9.7 keV energy were successfully focused onto an area of size 1.94 µm × 2.08 µm FWHM.

## Abstract 2

Microfocusing of hard X-ray free-electron laser pulses using Kirkpatrick–Baez mirrors at the nano-crystallography and coherent imaging hutch of the Pohang Accelerator Laboratory X-ray Free-Electron Laser facility is reported.

## 1. Introduction   

In the X-ray regime, X-ray free-electron lasers (XFELs) produce unprecedented levels of brilliance, excellent spatial coherence and ultrafast pulse durations (McNeil & Thompson, 2010 ▸). These unique properties provide opportunities to observe materials with high resolution, or facilitate radiation-damage-free and time-resolved studies using ultrashort pulse widths (Neutze *et al.*, 2000 ▸; McNeil & Thompson, 2010 ▸). In terms of XFEL facilities, the Linac Coherent Light Source (LCLS) (Emma *et al.*, 2010 ▸) and SPring-8 Ångstrom Compact free-electron LAser (SACLA) (Ishikawa *et al.*, 2012 ▸) facilities are both successfully operating hard XFELs. Recently, the Pohang Accelerator Laboratory (PAL-XFEL) (Ko *et al.*, 2017 ▸) and the European XFEL (Decking & Limberg, 2013 ▸) completed self-amplified spontaneous emission (SASE) commissioning and are now in operation. In addition, the SwissFEL (Milne *et al.*, 2017 ▸) is now preparing for XFEL lasing.

Construction on the PAL-XFEL began in 2010 and was completed in 2015. The first phase of the PAL-XFEL includes two beamlines: a hard XFEL beamline and a soft XFEL beamline. The hard X-ray beamline consists of a 10 GeV linear accelerator (700 m), undulator hall (250 m) and experimental hall (60 m) (Ko *et al.*, 2017 ▸). The hard X-ray experimental hall consists of two experimental hutches (EHs). In the first EH, which is called the X-ray scattering and spectroscopy (XSS) hutch, time-resolved experiments using the pump–probe technique are performed using primarily reflection geometry (Park, Eom *et al.*, 2016 ▸). In the second EH, which is called the nano-crystallography and coherent imaging (NCI) hutch, the most commonly performed studies are imaging studies that involve coherent X-ray diffraction imaging or serial femtosecond crystallography (Park, Kim *et al.*, 2016 ▸). These experimental instruments incorporate forward-scattering geometries and support time-resolved experiments, which can be used separately or in combination. In such studies, it is essential to generate a high photon flux density at the sample position using X-ray focusing optics. Various optical focusing devices, such as Fresnel zone plates (David *et al.*, 2011 ▸; Nilsson *et al.*, 2012 ▸), refractive lenses (Schropp *et al.*, 2013 ▸) and reflective mirrors (Yumoto *et al.*, 2013 ▸; Mimura *et al.*, 2014 ▸), are employed in other XFEL facilities. Among these, reflective mirrors achieve the highest focusing efficiencies over long working distances, and provide significant advantages in other applications. Thus, they were selected for use in our experimental devices. A microfocusing Kirkpatrick–Baez (KB) mirror system, which is a type of reflective mirror system, was developed for hard XFEL focusing applications at the PAL-XFEL. In this paper, we describe the main optical components for the hard XFEL beamline, the optical configuration of the microfocusing KB mirror system, and the current hard XFEL focusing capability at the PAL-XFEL.

## 2. Optical configuration of the focusing mirrors   

**Figure 1:**
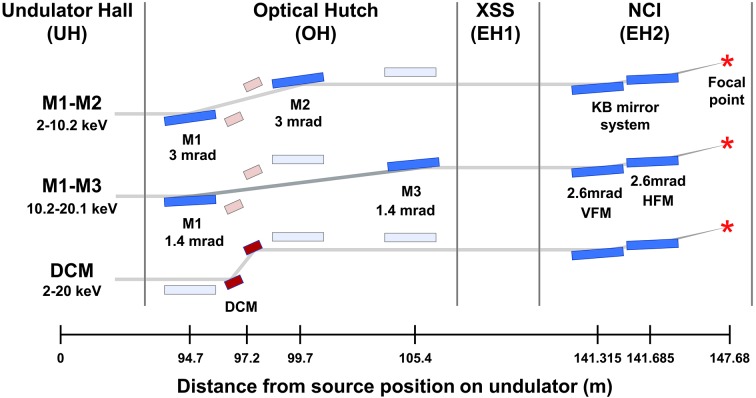
Schematic of the optical system of the NCI hutch at the hard X-ray beamline of the PAL-XFEL. The hard X-ray beamline includes three OMs (M1, M2 and M3) and a DCM at the OH. The KB mirror focusing system employs vertical and horizontal mirrors with focusing distances of 5.995 m and 5.365 m, respectively.

The PAL-XFEL hard X-ray beamline consists of an undulator hall (UH), optical hutch (OH) and two EHs. The microfocusing KB mirror system is located in the NCI hutch (Fig. 1 ▸). To transport the XFEL beam to the EHs, offset mirrors (OMs) or a double-crystal monochromator (DCM) in the OH are used for γ-ray shielding. The OMs and DCM are vertically offset by 30 mm and provide polychromatic or monochromatic X-rays, respectively. As a result, the XFEL beam from the UH enters the OH at a height of 1400 mm from the ground, and is delivered through the optical system at the OH to the EHs at 1430 mm. The XFEL beam passing through either the OMs or DCM follows the same path and moves parallel to the EHs.

The focused beam size that can be obtained using the developed focusing mirror system is approximately 2 µm. The optical configuration was designed based on the expected location of the beam source and the theoretical source size. The optical design parameters are listed in Table 1 ▸. The radiation size and divergence of the hard XFEL source of PAL-XFEL were calculated using the *GENESIS* (Reiche, 1999 ▸) code simulation with various accelerator parameters (Parc *et al.*, 2014 ▸). At a photon energy of 10 keV, the predicted source size and divergence are approximately 37.9 µm (FWHM) and 1.7 µrad (FWHM), respectively. The estimated incident beam size at the KB mirror is 280 µm. The length of the KB mirror is 600 mm, the mirror employs a quartz substrate, the grazing incidence angle of the mirror is 2.6 mrad, and the focusing mirrors have a large spatial acceptance of 1.53 mm vertically and 1.51 mm horizontally, which is large enough to reflect spatially full XFEL pulses. The distance from the expected XFEL source position in the undulator to the centers of the vertical and horizontal focusing mirrors (VFM and HFM, respectively) are 141.685 m and 141.315 m, respectively. The focal lengths of the VFM and HFM are 5.995 m and 5.365 m, respectively. The ideal focused beam sizes are approximately 1.6 µm (FWHM) and 1.4 µm (FWHM) at 10 keV in the vertical and horizontal directions, respectively, which are calculated by considering both the source size and the optical geometry. The long working distance of 5.065 m between the edge of the downstream mirror and the focal point is useful for combining various types of instruments with the focusing mirror system.

The effective area of the focusing mirrors is sagittally divided into two 10 mm-wide optical strip lines. For high reflectivity, one of the effective areas was coated with a 25 nm-thick carbon material, which has a theoretical reflectivity of greater than 99% at 2–11 keV. Another effective area was a bare quartz surface to minimize or eliminate the speckle pattern from the coating material. The carbon-coated area is intended for experiments requiring high photon flux, while the non-coated area is used for coherent diffraction imaging experiments requiring speckle-free XFEL pulses.

## 3. Evaluation of focusing performance   

To verify the theoretically calculated focused beam size, we directed X-rays through the developed focusing mirror and mirror manipulation system at the NCI hutch. The XFEL pulses used in this study were generated by a polychromatic X-ray passing through the OMs (*i.e.* a combination of M1 and M2) at the OH. The photon energy was 9.7 keV and the repetition rate was 10 Hz. The complete beam path and KB mirrors were operated in a vacuum of about 10^−8^ torr. Four-way slits were placed upstream and downstream of the KB mirrors to remove the halo and suppress parasitic scattering from the edge of the mirror. In addition, a Ce:YAG beam profile monitor and quadrant beam position monitors (QBPMs) were also installed upstream and downstream of the KB mirrors to monitor the beam position and intensity loss during reflection by the KB mirrors. In the QBPM, more than 99% of the X-rays were transmitted through a thin silicon nitride membrane, and the backscattered X-rays were measured using four photodiodes. The intensity loss caused by the KB mirrors was measured by the QBPMs to be about 5%. Considering that in our experiments we used a bare area of quartz substrate with a reflectance drop of about 5%, we were still able to confirm that most of the X-rays entered through the mirror aperture.

**Figure 2:**
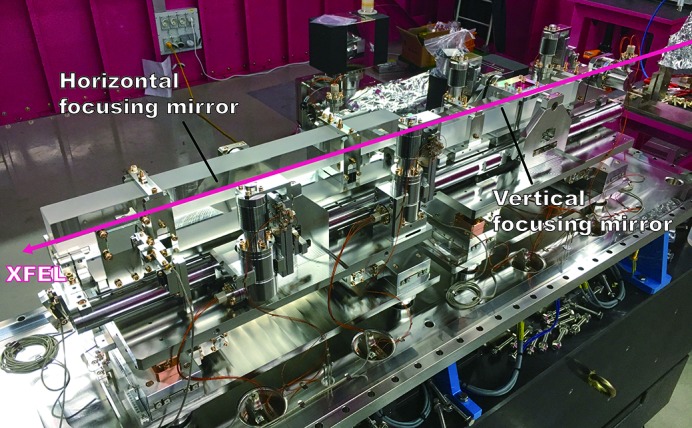
Photograph of the precision mirror manipulator with the elliptical VFM and HFMs at the NCI hutch at the PAL-XFEL.

In order to precisely align the focusing mirrors, the mirror manipulator shown in Fig. 2 ▸ was developed along with the focusing mirrors. The mirror manipulator has five degrees of freedom (*X*, *Y*, yaw, pitch and roll) for each VFM and HFM. The distance between the VFM and HFM, which is associated with the *Z*-translation, was fixed at 30 mm (Fig. 2 ▸). The motion precision of the mirror manipulators was measured based on the angle variation of the autocollimator and linear encoder. The linear encoder recognizes each motion inside the vacuum chamber, and the autocollimator measures the reflected value through the viewport outside the chamber. The motion test results of the KB mirror manipulator show that the resolution of the linear actuator for *X*- and *Y*-translation were 21.44 nm and 10 nm, respectively. The angular resolution of the pitch, roll and yaw were 22 nrad, 86 nrad and 30 nrad, respectively. As a result, all manipulator motions were suitable for use during KB mirror alignment.

**Figure 3:**
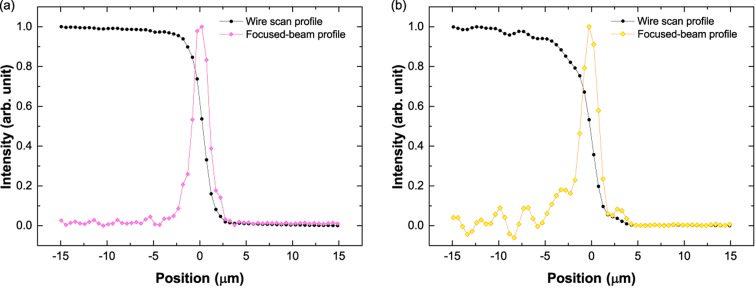
Typical focused-beam profiles measured horizontally (*a*) and vertically (*b*) with the wire scanning method. The measured beam size was 1.94 µm (FWHM) in the horizontal direction (*a*), and 2.08 µm (FWHM) in the vertical direction (*b*).

In order to measure the focused beam size, a wire scanning method was used with a 200 µm-diameter tungsten wire that was placed on a high-resolution motion stage at the focal point. A PIN photodiode placed behind the wire was also used to measure the beam intensity while scanning the wire. In this measurement procedure, a double-side polished single-crystal silicon attenuator was used to avoid ablation of the wire by the focused beam. The measured intensity profiles of the focused beam are shown in Fig. 3 ▸. Each point represents the averaged intensity of ten pulses. The focused beam size was determined to be 1.94 µm (H) × 2.08 µm (V) FWHM, which is in good agreement with the designed values.

## 4. Summary   

The main optical components and the microfocusing KB mirror system installed at the NCI hutch at the PAL-XFEL are described. The XFEL was focused at the target beam size (2 µm) through the developed mirror manipulator. In addition, this paper describes the optical configuration (polychromatic or monochromatic X-rays) that can be used in the NCI experimental hutch, and the diagnostic devices located upstream and downstream of the KB mirror system. These results will be useful for studies that will be performed at the NCI hutch.

## Figures and Tables

**Table 1:** Optical parameters of the focusing mirrors

	Vertical focusing mirror	Horizontal focusing mirror
Surface profile	Elliptical cylinder	
Substrate material	Quartz	
Surface coating	Carbon 25 nm (10 mm width) and non-coated (10 mm width)	
Mirror substrate size	600 mm × 50 mm × 50 mm (L × W × H)	
Effective mirror area	588 mm × 22 mm (L × W)	581 mm × 21 mm (L × W)
Grazing incidence angle	2.6 mrad	
Focal length (m)	5.955	5.365
Semi-major axis (m)	73.8	73.8
Semi-minor axis (mm)	75.8	71.8
Spatial acceptance (mm)	1.53	1.51
